# The RNA-seq transcriptomic analysis reveals genes mediating salt tolerance through rapid triggering of ion transporters in a mutant barley

**DOI:** 10.1371/journal.pone.0229513

**Published:** 2020-03-18

**Authors:** Sareh Yousefirad, Hassan Soltanloo, Seyedeh Sanaz Ramezanpour, Khalil Zaynali Nezhad, Vahid Shariati

**Affiliations:** 1 Department of Plant Breeding and Plant Biotechnolgy, Gorgan University of Agricultural Sciences and Natural Resources, Gorgan, Golestan, Iran; 2 Department of Genome Center, National Institute of Genetic Engineering and Biotechnology, Tehran, Iran; University of Delhi, INDIA

## Abstract

Considering the complex nature of salinity tolerance mechanisms, the use of isogenic lines or mutants possessing the same genetic background albeit different tolerance to salinity is a suitable method for reduction of analytical complexity to study these mechanisms. In the present study, whole transcriptome analysis was evaluated using RNA-seq method between a salt-tolerant mutant line “M4-73-30” and its wild-type “Zarjou” cultivar at seedling stage after six hours of exposure to salt stress (300 mM NaCl). Transcriptome sequencing yielded 20 million reads for each genotype. A total number of 7116 transcripts with differential expression were identified, 1586 and 1479 of which were obtained with significantly increased expression in the mutant and the wild-type, respectively. In addition, the families of WRKY, ERF, AP_2_/EREBP, NAC, CTR/DRE, AP_2_/ERF, MAD, MIKC, HSF, and bZIP were identified as the important transcription factors with specific expression in the mutant genotype. The RNA-seq results were confirmed at several time points using qRT-PCR for some important salt-responsive genes. In general, the results revealed that the mutant accumulated higher levels of sodium ion in the root and decreased its transfer to the shoot. Also, the mutant increased the amount of potassium ion leading to the maintenance a high ratio [K^+^]/[Na^+^] in the shoot compared to its wild-type via fast stomata closure and consequently transpiration reduction under the salt stress. Moreover, a reduction in photosynthesis and respiration was observed in the mutant, resulting in utilization of the stored energy and the carbon for maintaining the plant tissues, which is considered as a mechanism of salt tolerance in plants. Up-regulation of catalase, peroxidase, and ascorbate peroxidase genes has resulted in higher accumulation of H_2_O_2_ in the wild-type compared to the mutant. Therefore, the wild-type initiated rapid ROS signals which led to less oxidative scavenging in comparison with the mutant. The mutant increased expression in the ion transporters and the channels related to the salinity to maintain the ion homeostasis. In overall, the results demonstrated that the mutant responded better to the salt stress under both osmotic and ionic stress phases and lower damage was observed in the mutant compared to its wild-type under the salt stress.

## Introduction

Soil salinity is known as a major environmental stress limiting the growth and development of plants, resulting in a considerable reduction of crop productivity and yield [1]. Therefore, understanding the mechanisms involved in salinity tolerance can be effective in improving cultivars. Among all cereal crops, barley (*Hordeum vulgare* L.) is a salt-tolerant crop with significant economic importance in the world [2]. Salinity tolerance in barley is a complex quantitative trait comprising more than a hundred genes that may affect each other in different pathways [3, 4].

Plant response to environmental stress occurs via a series of physiological, cellular, and molecular mechanisms [5]. Such mechanisms include changes in morphology, anatomy, water relations, photosynthesis, hormones, toxic ion distribution, and biochemical adaptation such as the antioxidative metabolism [6, 7, 8]. Salt stress impacts the root system of plants in the first place by instigating osmotic stress in short term and then results in ion toxicity effects due to nutrient imbalance in cytosol via inducing ionic stress in long term [9].

Moreover, various plant responses have been observed in long term. Some plants show a higher tolerance compared to salt-susceptible ones under salinity conditions due to maintaining a high K^+^/Na^+^ ratio in the cytosol by various salt-tolerance mechanisms. However, these mechanisms rely on regulation and function of K^+^, and Na^+^ transporters, as well as H^+^ pumps, which generate the driving force for K^+^ and Na^+^ transport such as H^+^-ATPases and H^+^-PPases [2]. Some plants limit Na^+^ uptake (by salt exclusion) and/or reduce its cytosolic Na^+^ concentration (by sequestration of ions in the vacuoles via ion transporters), thus avoiding toxic effects on photosynthesis and other key metabolic processes [10]. In addition, salt stress contributes to oxidative stress, resulting in the generation of reactive oxygen species (ROS) (mainly hydrogen peroxide, H_2_O_2_) in various cell parts, such as chloroplast, mitochondria, and apoplast [11]. ROS production functions as a signal and regulator in plant development and is considered as a common plant response to all different environmental stresses [12]. Enzymatic antioxidants such as superoxide dismutase (SOD), catalase (CAT), ascorbate peroxidase (APX) and peroxidases (POX) are activated to maintain the balance between the rate of formation and removal of ROS to cope with salt stress [13]. Moreover, synthesis and accumulation of compatible solutes and maintaining water uptake are instrumental in osmotic adjustment, membrane and protein protection or ROS scavenging [14]. These responses will lead to restoration of cellular homeostasis, detoxification and therefore plant survival under salt stress [5]. Some main transporters involved in regulation of Na^+^ and K^+^ homeostasis and salt tolerance include SOS1 (salt overly sensitive), HAK, HKT (high-affinity potassium transporter) and NHX (Na^+^/H^+^ exchanger) [15].

Under saline conditions, plants generally activate the cascades of molecular networks involved in stress perception or sensing [16] and signal transduction [17] as well as induction of specific stress-related genes and their metabolites to survive [18]. Although some of the signaling pathways are specific, others may cross talk such as MAPK cascades and biotic signaling [19, 20]. Previous studies on various plant species demonstrated that cross talk included the complex networks of gene regulation [21, 22], which are influenced via plant hormones, such as ethylene (ET), jasmonic acid (JA) and abscisic acid (ABA) [21, 23], and transcription factors (TFs) [19, 24]. Cross talk also leads to changes in the expression of other genes responsible for osmoregulation and cell protection [19].

NADPH oxidase expression (RBOH) leads to an increase in ROS and MAP kinase as well as expression of *ACC* synthase, which in turn induces the activation of SOS1 antiporter and K^+^ channels in Arabidopsis under salt stress [25, 26]. Moreover, salinity stress induces the production of ethylene that may act as a downstream signal altering expression of other genes in various pathways related to salinity [27]. In general, knowledge about signaling pathways in short and long time periods is essential for understanding the regulation of different genes interconnected at several points under various stresses [5].

The RNA-Seq technique utilizes next-generation sequencing (NGS) and is regarded as an efficient tool for sequencing the cDNA derived from an RNA sample, which eventually produces millions of short reads. This technique also analyzes the differentially expressed (DE) genes and biological processes under salinity conditions [19]. Moreover, the RNA-Seq method has considerable potential to generate high-resolution transcriptome maps sensitive enough to display transcripts even with low levels of expression [19, 28]. The present study utilized RNA-Seq analysis in two genotypes including a salt-tolerant mutant line “M4-73-30” and its wild-type cultivar “Zarjou” at an early time (six hours) after exposure to salinity stress in order to evaluate transcriptome analysis of salt-related genes, physiological differences and processes. The result may improve the current understanding about tolerance mechanisms and effects of the mutation in barley in response to salt stress.

## Materials and methods

### Plant materials and salt stress conditions

Two barley (*Hordeum vulgare* L.) genotypes including a salt-tolerant mutant line “M4-73-30” and its wild-type cultivar “Zarjou” were used for the present study. These genotypes had been provided by Seed and Plant Improvement Institute, Karaj, Iran [29, 30]. A salt-tolerant mutant genotype was produced by the gamma irradiation approach and evaluated for salt tolerance through field trials. This genotype had been previously introduced as a salt-tolerant cultivar called "Roodasht" by Seed and Plant Improvement Institute, Karaj, Iran [31]. Previous studies [29, 31, 30] revealed that a salt-tolerant mutant had a higher ability to tolerate salt stress than its wild-type genotype by physiological response related to salinity tolerance.

Uniform seeds of both genotypes were surface-sterilized using 5% sodium hypochlorite for five minutes and were rinsed with distilled water for 10 minutes. Sterilized seeds of each of genotype were germinated on moistened germination paper in 15 cm diameter glass Petri dishes in an incubator at 22±1°C under dark conditions for four days. After germination, the seedlings were transferred into a half-Hoagland nutrient solution at pH 6.0 [32]. The plants were grown in controlled conditions at 25/22°C day/night, 16 hours light duration, and 70% humidity in a greenhouse at Gorgan University of Agricultural Sciences and Natural Resources, Gorgan, Iran. Afterward, the seedlings were exposed to salt stress at two-leaf stage by adding NaCl at a concentration of 300 mM to the nutrient solution including CaCl_2_ to maintain Na^+^/Ca^2+^ ratio of 10:1 on a molar basis. The root and shoot samples were harvested separately in three replications (including six plants for each replication) after three, six, 12, 24, 48, 72, and 96 hours of exposure to the salt stress. The plant seedlings grown only under normal Hoagland nutrient solution were used as the control (0). All samples were immediately frozen in liquid N_2_ and stored at −80°C for further use.

### RNA isolation

The total RNAs were extracted separately from the root and shoot samples by the Biozol method (BioFlax, Japan) [29]. The RNA quality was checked by 1% agarose gel electrophoresis and 30 μg was used for RNA-Seq. Afterward, the RNA samples were shipped to Beijing Genomics Institute (BGI), Hong Kong, China for deep sequencing and generation of datasets. The Agilent 2100 Bioanalyzer System and RNA 6000 Nano were used to generate information on the RNA concentration, the ribosomal ratios and the RNA integrity number (RIN) [33].

### Next-generation RNA sequencing

The transcriptome analysis was evaluated through mRNA-seq method and using Truseq kit on Illumina HiSeq^TM^ 2500 platform as paired-end at read length of 150 nt. Raw reads were subjected to quality control using the Trimmomatic software (Version 0.36) 62 to filter out adaptor and low-quality nucleotide/sequences. After trimming, FastQC (http://www.bioinformatics.babraham.ac.uk/projects/fastqc/) was used to examine the characteristics of the libraries and to verify the trimming efficiency. The high-quality filtered reads were used for downstream analyses. Filtered reads were aligned against the barley genome as the reference using Hisat2 package after downloading the *Hordeum vulgare* transcript sequences from the NCBI database (http://www.ncbi.nlm.nih.gov/unigene). Read counts were calculated using cufflinks and htseq, and outputs were used for differential expression analysis by Cuffdiff and EdgeR packages. To obtain the differentially expressed genes (DEGs), a threshold of adjusted p value≤ 0.0001 and an absolute value of log2FC≥ 2 were applied. The GO category enrichment analysis for DEGs was performed using goseq and AgriGO (version 2.0). Therefore, GO analysis was described in three categories: biological process, molecular function, and cellular component for the upregulated DE transcripts under the salt stress. The pathway enrichment analysis was carried out by in-house scripts and using KEGG and Reactome databases. Significant pathways were identified by using Fisher’s exact test and corrected P values < 0.001.

### Validation of RNA-seq data by real-time PCR

The validation of the RNA-Seq data for four selected genes was performed using real-time PCR with the *TEF* and *α-tubulin* as a house-keeping gene. Primers were designed with Primer 3 software (www.embnet.sk/cgi-bin/primer3_www.cgi) based on 3' untranslated region (3'-UTR). The primer names and the sequences used for primer designing are given in Table 1. For qRT-PCR, the quantity of mRNA corresponding to each gene was measured by SYBR Green. The PCR mixture consisted of 9.9μl 2X SYBR Bio Pars (SBB, Iran) PCR Master Mix, 0.5μl DMSO, 0.5μl of each gene-specific forward and reverse primers (10 pmol), 0.2μl *Taq* enzyme, and 3μl of the diluted cDNA in a final volume 17μl with double distilled water. Forty PCR cycles for each gene product included denaturation at 95°C for five min and 10s at 95°C, annealing at 58–60°C for 10s and extension at 72°C for 20 s and five min at 80°C for one cycle. The specificity of the amplicons was checked by melting curve analysis (10s at 55°C) after 81 cycles. α-tubulin as a reference gene by the comparative Ct (2^−ΔΔCt^) method was used to calculate the relative expression (Livak and Schmittgen, 2001)[34]. Expression pattern analysis of *Rboh*, *ACC synthase*, *HAK* and *HVP* genes were detected by qRT-PCR in contrast to *α-tubulin*, a reference gene to normalize data, in the root and shoot samples under salt stress. PCR for each sample was performed in three technical and biological replications. Relative expression levels were computed using the following formula presented by Pfaffl and Hegeleit [35]. The ratio analysis between the amount of the target gene and the housekeeping reference genes was performed by the REST software [36].

**Table 1:** The name and sequence of specific primers used for the real-time PCR amplification.

Primer name	Sequence		Tm	GC%	Product size (bp)
*RBOH*	Forward	5َ-GCGGGTCTACTTCTACTGGT-3´	55	55	181
	Reverse	5َ-CTGCAGCATCACCACCATG-3´	57.8	57.9	
*ACC Synthase*	Forward	5َ-TTGTGCAGATGATGTTCGGG-3´	58.5	50	158
	Reverse	5َ-GCACCGCATGTACTCGATC-3´	58.7	57	
*HVP1*	Forward	5َ-CGTCGCTCAACATCCTCATC-3´	58.8	55	183
	Reverse	5َ-GGTTGACCTAAGCCTCCACT-3´	59	55	
*HAK*	Forward	5َ-TGCTCAAAGTCGGGATCACA-3´	59.3	50	199
	Reverse	5َ-AAAGAACACCCCTCCCTACC-3´	58.6	55	
*α-tubulin*	Forward	5َ-AGTGTCCTGTCCACCCACTC-3´	60.1	60	289
	Reverse	5َ- ATTCAGAGCACCGTCAAACC-3´	57.5	50	

## Results

### Analysis of RNA-seq datasets

In this research, the results of RNA-seq analysis indicated that a total number of 3184 up-regulated transcripts with differential expression were identified in salt-tolerant mutant “M4-73-30” and its wild-type “Zarjou” genotypes at seedling stage after six hours of exposure to salt stress (300 mM NaCl). In total, 1586 and 1479 transcripts were identified in the salt-tolerant mutant and wild-type genotypes, respectively. These DE genes of the gene ontology analysis were assigned to three main categories; molecular function, biological process, and cellular component. The results of gene anthology analysis demonstrated that the greatest number of genes involved in final molecular functions in salt-tolerant mutant genotype were calcium ion binding, peroxidase activity, transcription factor, and serine-type endopeptidase inhibitor activity (S1 Fig). Moreover, the differentially expressed genes related to the final biological process in this genotype included potassium ion transport, cation transmembrane transport, phenylalanine catabolic process, and regulation of transcription DNA-templated (S2 Fig). Up-regulated genes detected in parts of the apoplast and endoplasmic reticulum membrane of the salt-tolerant mutant genotype were involved in the cellular component (S3 Fig).

On the other hand, the highest transcript abundance of the genes involved in the final molecular functions in the wild-type genotype included electron transporter in photosynthesis and cytochrome-c oxidase activity (S4 Fig). In addition, the differentially expressed genes involved in biological process in the wild-type genotype included the response to ROS, photosynthetic electron transport in photosystem I and glycine metabolic process as well as regulation of photosynthesis (S5 Fig). Due to their involvement in cellular component, the differentially upregulated genes were in mitochondria and chloroplast of the wild-type genotype (S6 Fig).

**Fig 1:**
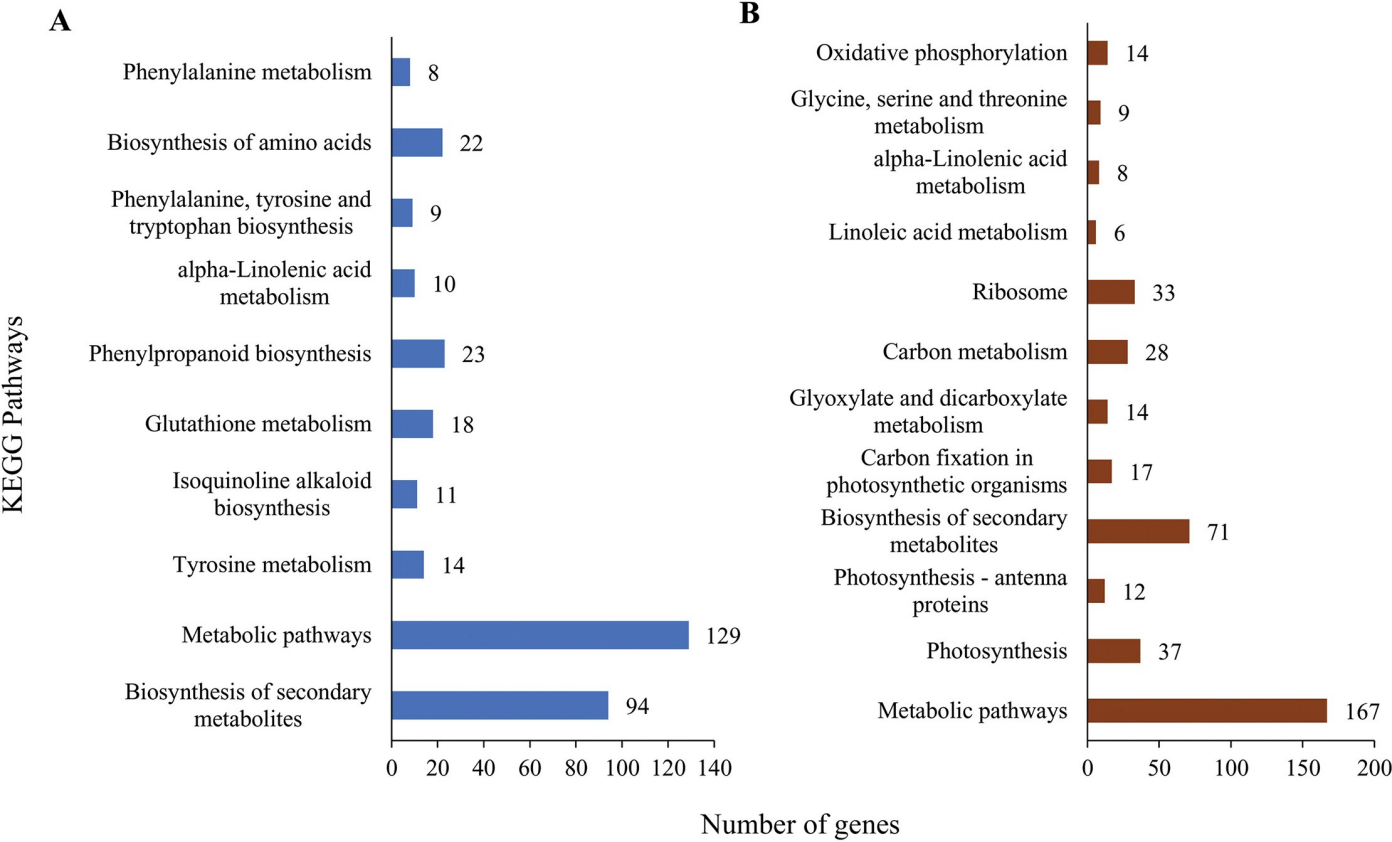
Number of differentially expression genes under salt stress. (A) M4-73-30 (B) Wild-type.

**Fig 2:**
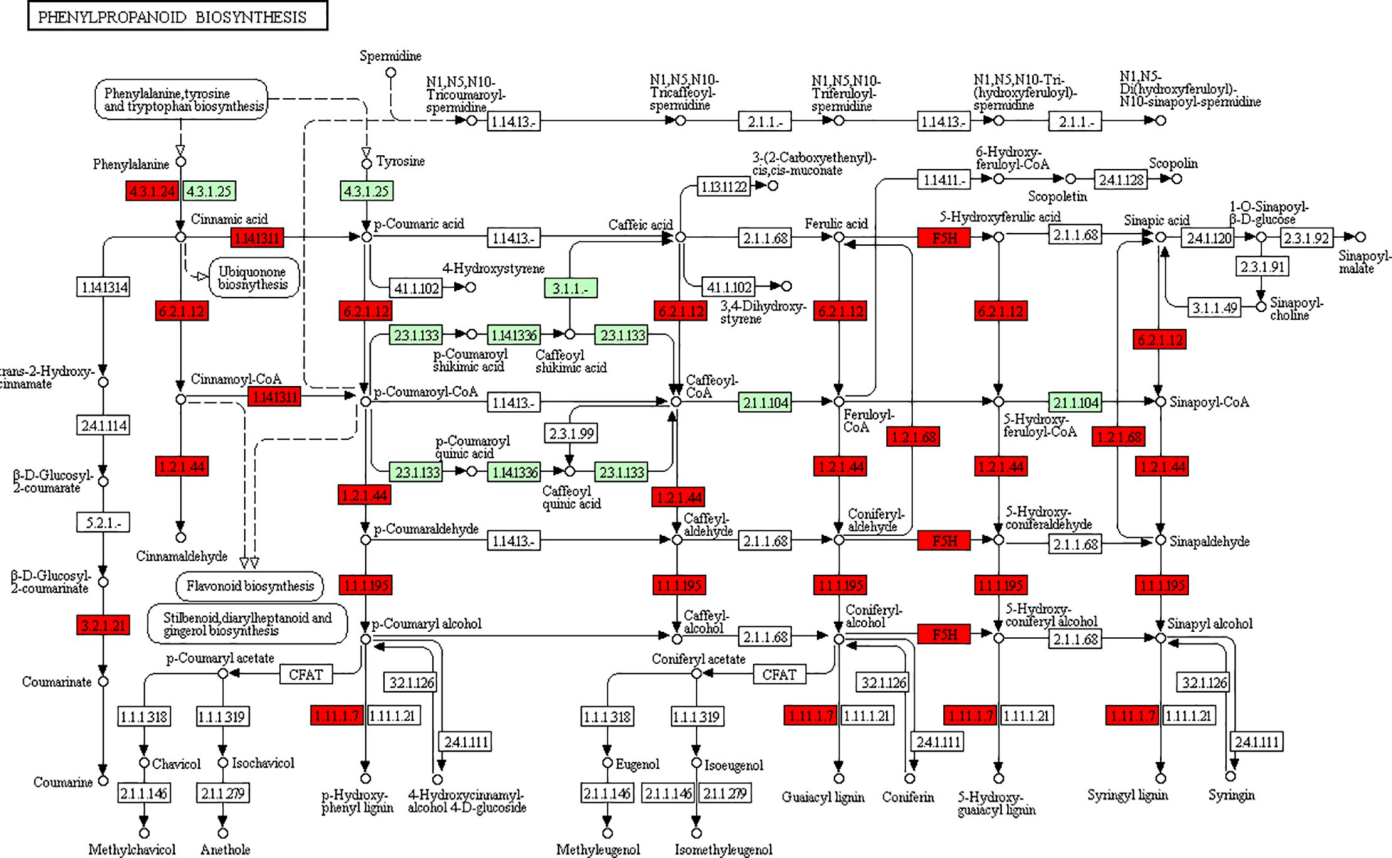
KEGG significantly phenylpropanoid biosynthesis pathway in the salt-tolerant mutant genotype under salt stress. It represents one of the KEGG pathway for up-regulated genes in response to 300 mM salt (six hours of exposure) in the salt-tolerant mutant genotype.

All the DE transcripts were mapped to reference standard pathways in the Kyoto Encyclopedia of Genes and Genomes (KEGG) (http://www.genome.ad.jp/kegg/) to identify the different pathways activated following six hours of exposure to salt stress in the two barley genotypes. In this study, 95 pathways were identified in the salt-tolerant mutant genotype in total that 10 of which had the significant DE, which in turn were comprised of 338 up-regulated genes. Moreover, metabolic pathways, biosynthesis of secondary metabolites and phenylpropanoid biosynthesis included the highest number of genes in the salt-tolerant mutant genotype (Fig 1A). KEGG phenylpropanoid biosynthesis pathway in the salt-tolerant mutant genotype under salt stress is represented in Fig 2. On the other hand, the results of KEGG analysis indicated that 12 out of 96 identified pathways included 416 differentially expressed genes in the wild-type genotype. Furthermore, pathways with the highest number of genes were involved in the metabolic pathways, the biosynthesis of secondary metabolites, and photosynthesis in the wild-type genotype (Fig 1B).

Furthermore, among all these genes with differential expression, 11 important families of transcription factors such as WRKY, ERF, AP_2_/EREBP, NAC, Cytochrome P450, CTR/DRE, AP_2_/ERF, MAD, MIKC, HSF, and bZIP 91 genes were found in the salt-tolerant mutant genotype. Also, three families of transcription factors including TFIID, HSP, and Cytochrome P450 were identified in the wild-type genotype which were comprised of 24 genes relating to salt stress response.

### The expression pattern of genes involved in Na^+^ homeostasis under salt stress

**Fig 3:**
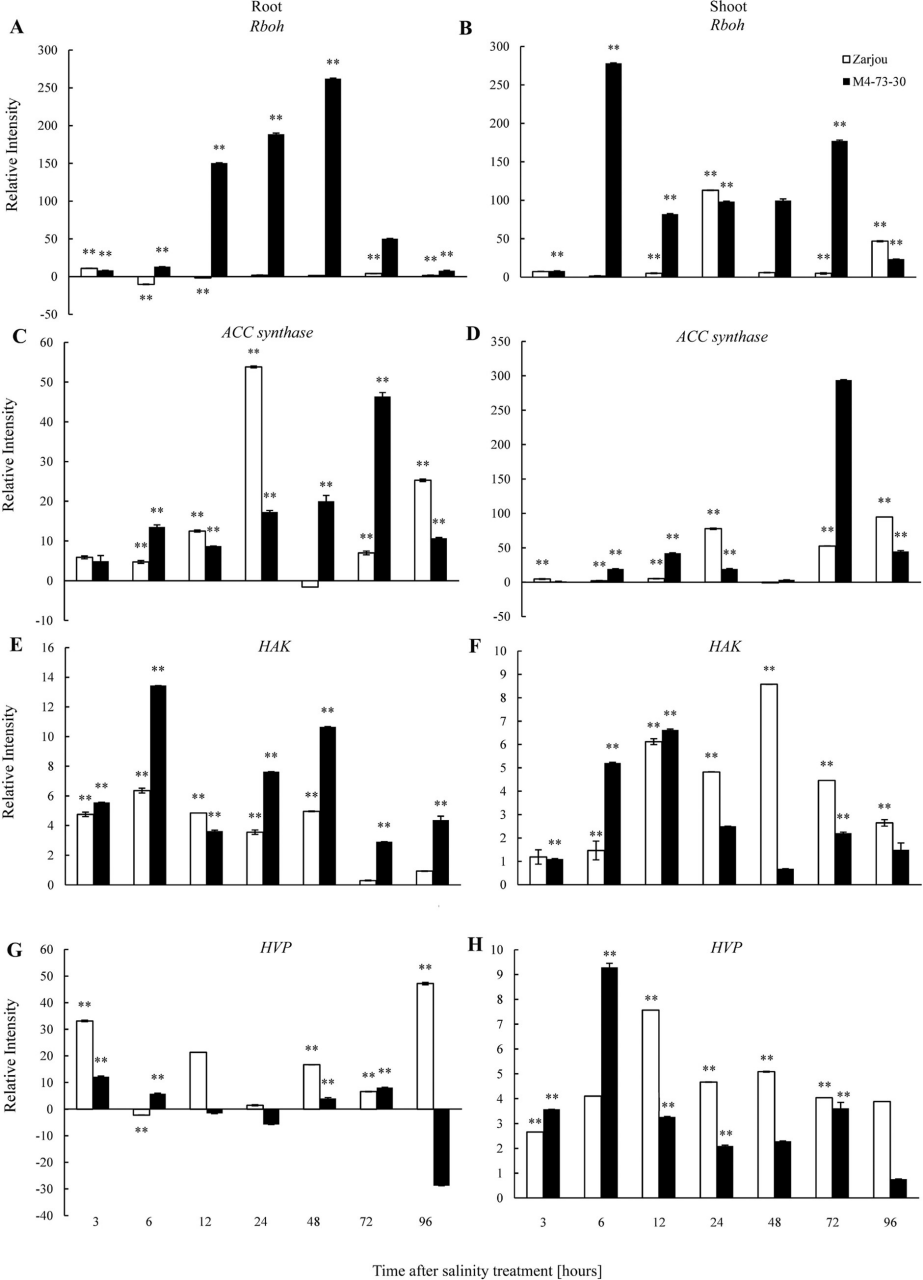
The expression pattern of genes in the salt-tolerant mutant genotype and its wild-type in contrast to control (0). (A, B) *Rboh* (C, D) *ACC synthase* (E, F) *HAK* (G, H) *HVP*. ** indicates significant difference at *P* ≤ 0.01.

Expression pattern analysis of *Rboh* (Fig 3A and 3B), *ACC synthase* (Fig 3C and 3D), *HAK* (Fig 3E and 3F), and *HVP* (Fig 3G and 3H) genes in the shoot and root samples of the salt-tolerant mutant “M4-73-30” and its wild-type “Zarjou” compared to the control (0) at different time points (3, 6, 12, 24, 48, 72, and 96 hours after exposure to salt stress) were evaluated by qRT-PCR technique in contrast to *α-tubulin* as a house-keeping gene under 300 mM NaCl (Fig 3).

The gene expression pattern of *Rboh* was increased significantly and reached its maximum level 48 hours after exposure to salt stress compared to the control (0) in the root sample of salt-tolerant mutant genotype. Also, this considerable increase in the root sample of the mutant genotype was approximately 250-folds higher than that of the wild-type genotype. Afterward, a decreasing trend was observed until 96 hours. After a sudden significant increase at six hours (more than 280-fold), the gene expression in the shoot sample of the salt-tolerant mutant genotype showed a moderate gradual increase until 72 hours. On the other hand, the expression level of *Rboh* gene in the shoot sample of the wild-type genotype had the highest level at 24 and 96 hours. However, the expression of this gene in the root and shoot samples of the wild-type genotype was not detected as a regular trend in contrast to those of the mutant genotype (Fig 3A and 3B).

The gene expression of *ACC synthase* up-regulated until 72 hours and then down-regulated at 96 hours in the root sample of the salt-tolerant mutant genotype. Moreover, the expression pattern of this gene in the shoot of mutant genotype was approximately similar to that of the (except at 24 hours and 48 hours). Meanwhile, the expression pattern of this gene in the root and shoot samples of the wild-type were similar, so that the highest gene levels were observed at 24 hours and 96 hours of exposure to the salt stress (Fig 3C and 3D).

The gene expression of *HAK* in the root sample of the salt-tolerant mutant genotype considerably increased at six hours compared to the control (0), which also showed a 2-fold increase in contrast to that of the wild-type genotype. The increase in expression of this gene was also observed at 24 hours and 48 hours after exposure of the salt stress. Furthermore, the expression level of this gene in the shoot sample of the salt-tolerant mutant genotype increased at six hours and continued until 12 hours, at which reached its highest level. However, the highest level of this gene expression in the shoot of the wild-type genotype was observed at 48 hours compared to that of the salt-tolerant mutant genotype (Fig 3E and 3F).

The expression level of *HVP* gene in the root and shoot samples of the salt-tolerant mutant genotype generally increased at 3 hours and 6 hours after exposure to salinity conditions, respectively. Furthermore, the highest amount of expression in this gene in the root and shoot samples of the wild-type genotype was observed at 96 hours and 12 hours after application of salt stress compared to the control (0) (Fig 3G and 3H).

**Fig 4:**
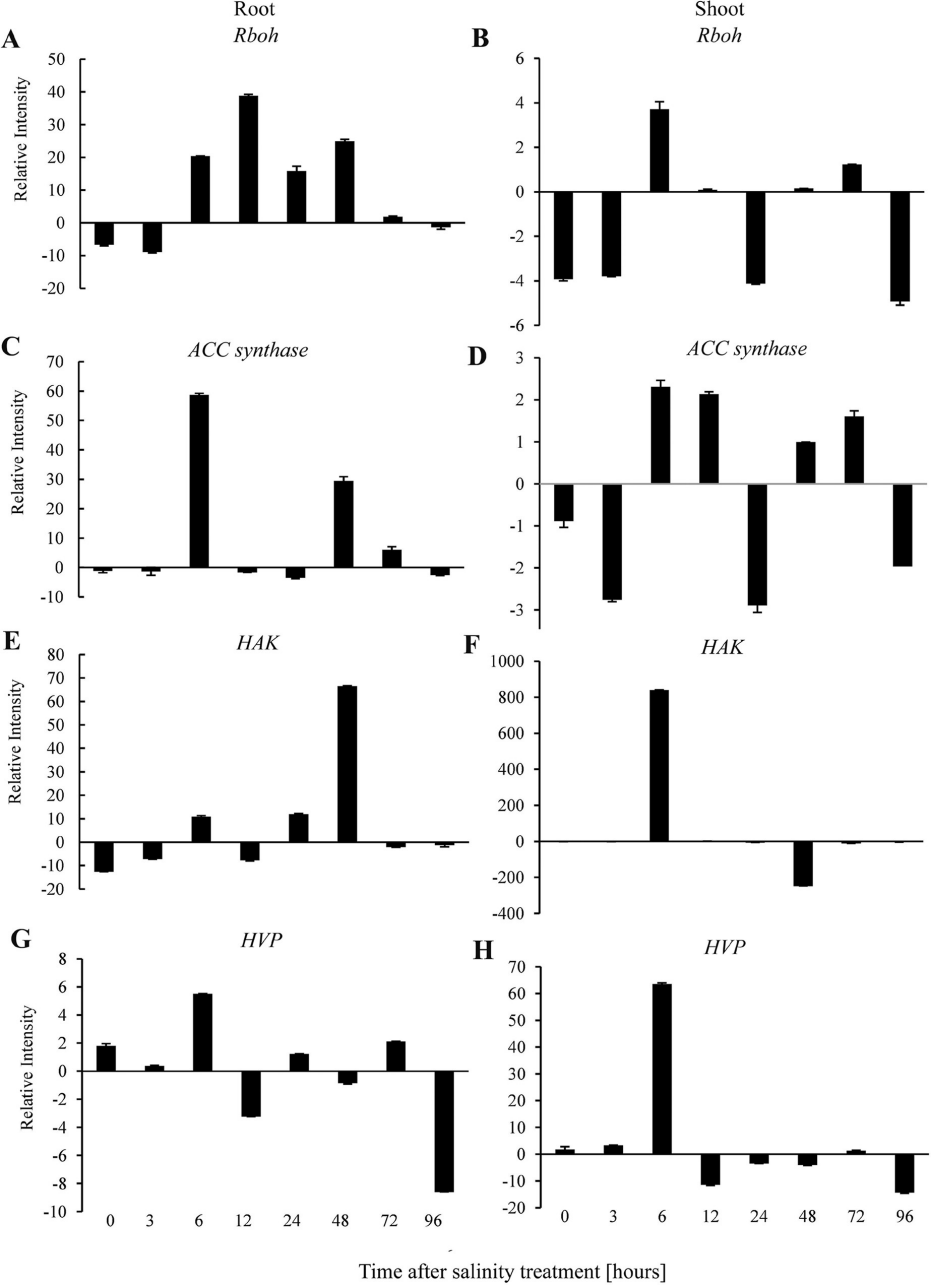
The expression pattern of genes in the salt-tolerant mutant genotype in contrast to its wild-type as the control. (A, B) *Rboh* (C, D) *ACC synthase* (E, F) *HAK* (G, H) *HVP*.

The gene expression analysis of *Rboh* (Fig 4A and 4B), *ACC synthase* (Fig 4C and 4D), *HAK* (Fig 4E and 4F), and *HVP* (Fig 4G and 4H) in the root and shoot samples of the salt-tolerant mutant genotype compared to its wild-type genotype at different time points under the salt stress is shown in Fig 4. The interesting parallel of the up-regulation of *Rboh*, *ACC synthase*, *HAK*, and *HVP* genes were identified in the short term (six hours) in the root and shoot samples of the salt-tolerant mutant in contrast with those of the wild-type genotypes. However, the increase in expression of *Rboh* gene was detected at 12 h and 24 h as well. Furthermore, the expression of *Rboh*, *ACC synthase*, *HAK* and *HVP* genes in the shoot of a salt-tolerant mutant genotype in comparison to that its wild-type genotype was parallel at six hours after exposure to NaCl which reached its maximum level (Fig 4).

## Discussion

Plants utilize various strategies to tolerate salinity conditions. Plants require a high cytosolic K^+^/Na^+^ ratio in their cytoplasm to survive under salt stress. Previous studies in various crop species such as barley, rice and maize indicated that minimizing Na^+^ accumulation or the ability to maintain K^+^ in the shoot under salinity has been positively correlated with salt tolerance [37, 38]. Under salinity stress, some tolerance mechanisms are evolved in plants to regulate K^+^ and Na^+^ to maintain cellular homeostasis under salinity stress. Restriction of Na^+^ uptake, Na^+^ exclusion to the soil and compartmentation of Na^+^ from cytosol to vacuoles and control of Na^+^ loading in xylem are crucial mechanisms for salinity tolerance [39, 40]. Many of these salt tolerance mechanisms depend on H^+^, K^+^ and Na^+^ transporters, such as SOS1, HKT, HAK and NHX to maintain the cellular ionic homeostasis for salt tolerance [41].

Salinity stress reduces soil water potential, and thus, induces water deficit and rapidly transmits a water deficit signal from root to shoot and finally contributes intracellular turgor reduction [41]. Therefore, salinity results in a reduction in stomatal conductance, the most significant response that immediately occurs after plant exposure to salt. Due to stomatal closure, salinity stress reduces the rate of transpiration (water loss) limiting a load of toxic ions within the transpiration stream in plant and photosynthesis (CO2 uptake) and hence, inhibits carbon fixation and accumulation of the ions in the shoot part of the plant affecting plant growth [42, 16, 43]. These events have been reported in several plant species [44, 45]. Koyro [46] showed that a decrease in stomatal conductance acts as an adaptive mechanism to salinity via maintaining salts at subtoxic levels.

Cytosolic calcium is a second messenger in signalling of abiotic stresses such as salinity. The initial increase in cytosol Ca^2+^ could be due to the production of ROS (mainly H_2_O_2_) sourced by the membrane NADPH oxidases (encoded by RBOH) which are involved in signaling during stress [47]. The results of this study showed that the gene expression of *Rboh* was significantly up-regulated in the root and shoot samples of salt-tolerant mutant genotype in comparison to its wild-type genotype in short term (six hours) after exposure to salt stress. Moreover, ROS increase leads to higher cytosol Ca^2+^ concentration resulting in the regulation of ion homeostasis via the activity of SOS1 antiporter, MAP kinase, SOS2 and CBLs [15]. Overexpression of these genes in the salt-tolerant mutant genotype at the same time (six hours), had been observed in the present and previous study [30].

Drerup et al, [48] demonstrated that RBOH in Arabidopsis can be activated by CIPK26/CBL1/9. In this study, the parallel up-regulation was shown in the salt-tolerant mutant genotype at six hours after exposure to salinity in comparison to its wild-type genotype. ROSs function as an important signaling molecules in adaptive and developmental responses of plants to abiotic and biotic stresses which can activate several signal pathways, such as hormonal signaling networks to enhance the salinity tolerance [49]. In addition, ROSs activate K^+^ ion channels leading to maintenance of cellular homeostasis [50, 51].

Chung et al, [25] showed that the activity of salt stress-induced SOS1 transcript requires RBOH through increment of Ca^2+^ concentration in the cytosol. Ca^2+^ is sensed by CBL4 (SOS3) protein which produces the SOS3/SOS2 complex after interaction with the serine/threonine protein kinase CIPK24 (SOS2). This complex activates the plasma membrane Na^+^/H^+^ antiporter SOS1 for Na^+^ efflux from cell to control ion homeostasis [52, 53, 30]. On the other hand, SOS3/SOS2 complex induces the activity of ion transporters such as NHX1 to compartmentalize Na^+^ from the cytoplasm to the vacuoles [54]. The driving force for this transporter is provided by two main vacuolar H^+^-pumps; H^+^-ATPase and H^+^-pyrophosphatase (V-PPase) indicating that these pumps have essential roles in response to salt conditions [55, 56]. In our previous study, the overexpression of the parallel genes (*SOS1-3*) in SOS pathway, *NHX1* transporter and *HVA* pump were observed in the salt-tolerant mutant genotype in contrast with wild-type genotype at 6 h after exposure to salinity stress [30].

Under salt stress, NADPH oxidase leads to the generation of ROS resulting in MAP Kinase activity, which is linked to salt tolerance [57]. MAPK superfamily, a member of the serine/threonine kinases, acts as a key player in some of the critical roles in plant signaling networks and is tolerant to various stresses including drought and salinity [58]. Moreover, many studies have indicated that in addition to MAPK pathway activity, salinity stress also induces hormones production and signals, such as ethylene in plants [59]. The plant hormones such as ethylene have a major role in plant development throughout germination, growth, and response to stress conditions. Previous studies have indicated that ROS are essential for transduction of ethylene signal in regulation of Na^+^ and K+ homeostasis to initiate the tolerance [60]. Moreover, it is shown that salt tolerance was induced by ethylene in Arabidopsis [61, 62]. Ethylene production acts as a mediator component in response to stress conditions in barley. To produce ethylene, S-adenosyl-L-methionine is converted to ACC by ACC synthase (or ACS). Finally, ACC is modified by ACC oxidase (or ACO), which can influence the expression of another set of genes [63]. Salt and osmotic stress induced the conversion of ACC to ethylene in the halophyte *Allenrolfea occidentalis* [64]. Ethylene binding to the receptor which interacts with Constitutive Response (CTR), initiates a transcriptional cascade and downstream ethylene responses [65]. Li et al, [66] demonstrated that ethylene production and activity of ACO were significantly increased in cucumber seedlings under salinity stress (75 mM NaCl). Moreover, the ethylene-responsive element binding factor (ERF) was vital in cotton under stress conditions [67]. Overexpression of sugarcane and soybean ERFs in tobacco conferred tolerance to high salinity stress (200 mM) [68]. Regarding parallel overexpression of these genes at hormonal pathway networks of ethylene production, these mechanisms were observed in the salt-tolerant mutant genotype after six hours of exposure to salt stress and enhanced the salinity tolerance.

Moreover, potassium (K^+^) is an essential factor in osmotic processes for resistance to salinity and drought. K+ decreases the toxic effects of Na^+^ and maintains high K^+^/ Na^+^ ratio in shoots and especially in leaves, which is important in glycophytes for improved salinity tolerance [69]. On the other hand, the production of ROS depends on K^+^ availability. Reduction in K^+^ content is associated with increased activity of enzymes involved in detoxification of H2O2. Therefore, the increase in K^+^ content reduces ROS via limiting the membrane NADPH oxidase which results in salinity tolerance [70]. K^+^ channels are vital to maintain and support plant development and growth. Researchers have categorized six gene families, including three channel families and three transporter families (HAK/KUP/KT, HKT, and CPA) [71, 72].

The main transporter of salt stress tolerance is HKT antiporter [16, 73]. HKT transporters have two Sub-families of which sub-family1 transporters are only permeable to Na^+^, whereas sub-family2 transport both K^+^ and Na^+^ [74]. HKT1;5 is a Na^+^ selective transporter, expressed in the plasma membrane of parenchyma cells surrounding xylem vessels [75, 76]. HKTs are involved in Na^+^ long-distance translocation by contributing to Na^+^ unloading through the xylem, preventing excessive accumulation of Na^+^ in leaves [77]. The up-regulation of the HKT1 transporters activity leads to a decrease in leaf Na^+^ content [78, 79]. Therefore, it increases the ability of control of K^+^/Na^+^ homeostasis for salinity tolerance [80]. It is revealed that *OsHKT1;5* reduced the root to shoot delivery of Na^+^ and increased salinity tolerance in rice [81, 82, 80]. In the present study, the gene expression of HKT1;5 was up-regulated at 6 h after exposure to salt stress in the salt-tolerant mutant genotype. In general, in parallel of increase in *Rboh* gene expression, the up-regulated genes of *MAPK*, *ACC synthase*, *HAK*, *HVP* and *HKT* were observed at the same time point (6 h) of exposure to salt stress in the salt-tolerant mutant genotype in comparison to the wild type genotype, which represented in Fig 4.

Furthermore, ethylene causes the preservation of cellular K^+^ via an increase in transcript level of *AtHAK* transporter in Arabidopsis under salt stress [15]. Under salt stress, HAK transporter plays a key role in K^+^ uptake through the root, as well as K^+^ long-distance transport through loading and unloading in the vascular tissue [83, 84]. Shin and Schachtman (2004) revealed that expression of *HAK5* gene in Arabidopsis root depended on RBOH activity and ROS. Moreover, *HAK5* activity in Arabidopsis roots is regulated by CIPK23, CBL1, CBL8, CBL9, and CBL10 proteins [85, 53]. In the current research, an increase in gene expression of *HAK* in the root and shoot samples of the salt-tolerant mutant genotype was considerably observed in the root sample at 6, 24 and 48 hours after exposure to salt stress in contrast to its wild-type genotype.

TPK1/KCO1 channel, another important family of K^+^ channels, have been localized in the vacuolar membrane. This channel contains the binding sites for Ca^2+^ and 14-3-3 proteins involved in the K^+^ transport from vacuole to cytosol to contribute a favorable Na^+^/K^+^ ratio and ion homeostasis [72, 53]. In the present study, the transcript level of TPK1/KCO1 gene and 14-3-3 protein was up-regulated after 6 h of exposure to salinity in the salt-tolerant mutant genotype in comparison with its wild type genotype.

Salt stress leads to production of ROSs and eventually induces some enzymatic antioxidants to remove them for salinity tolerance [13]. In this research, the higher expression of peroxidase was observed to remove H_2_O_2_ content in the salt-tolerant mutant genotype in comparison to its wild-type genotype after 6 h of exposure to salinity. Under salt stress, APX is one of the most significant antioxidant enzymes in plant cells and plays an essential role in the control of ROS levels regulated by redox signals and H_2_O_2_ [86]. Many researchers reported an increase in APX activity in response to abiotic stresses such as salinity, drought, chilling, and metal toxicity [87, 88]. Moreover, APX has a much higher affinity for H_2_O_2_ than CAT, which renders them efficient scavengers for H_2_O_2_ at high concentration under stress conditions [89]. Therefore, due to more accumulation of H_2_O_2_ in the wild-type genotype, the gene expression of enzymatic antioxidants, such as CAT, POX, and APX for ROS scavengers were significantly up-regulated in contrast to salt-tolerant mutant genotype in short term, i.e. six hours after exposure to the salt stress. The results of this study were similar to report of Kiani et al, [29]. Furthermore, the rate of evaporation via transpiration stream and H_2_O_2_ production via photosystems (I and II) were reduced due to stomatal closure, which is an important mechanism of salt tolerance [90, 16].

There are reports that salt stress induces MAPK pathway as well as activation of transcription factors such as WRKY in Arabidopsis [91]. Transcription factors are the most important regulators that control a wide range of gene expressions in different signaling pathways through binding to the specific cis-acting element in the promoters of genes [92]. Among all transcription factors, bZIP, WRKY, MYB, CTR/DRE, AP2, NAC, C2H2 zinc finger gene, and DREB families demonstrate high expression levels and comprise a large number of stress-responsive members [93, 94]. In this research, the results of mRNA-seq analysis revealed that the expression levels of important transcription factors were increased in the salt-tolerant mutant genotype in comparison to its wild-type genotype at six hours after exposure to the salinity stress. These transcription factors included WRKY, ERF, bZIP, AP_2_/ERF, NAC, AP_2_/EREBP, Cytochrome P450, CTR/DRE, MIKC, MAD, and HSF. Many researchers demonstrated that transcription factors, such as WRKY and NAC play key roles in ROS signaling pathways in response to stresses, resulting in salt tolerance in Arabidopsis [95, 96]. Nakashima et al, [97] revealed that the up-regulation of a NAC transcription factor in both rice and wheat plays a vital role in salt tolerance. Moreover, Song et al, [98] reported that NAC overexpression is induced by ROS (H2O2) in rice under salinity stress, which may regulate the synthesis and accumulation of components such as proline, sugar, and LEA proteins that play important roles in tolerance to stress.

The RNA-seq as high-throughput sequencing of cDNA has been the most important powerful tool for analysis during these decades. Recently, a genome sequence and transcript profiling of barley has been reported for the stress-responsive genes [19, 2]. In this research, sequencing of the cDNA samples of salt-tolerant mutant and its wild-type genotypes at an early time point (six hours) after exposure to the salt stress (300 mM NaCl treatment) yielded about 20 million reads for each genotype. Furthermore, a total number of differential expression transcripts included 7116, 1586, and 1479 DE transcripts with significant over-expressions were obtained in the salt-tolerant mutant and its wild-type genotypes, respectively.

Various researches demonstrated that the tolerant plants displayed a decreased respiratory rate, whereas this attribute increased in sensitive plants. Certain halophytes show a decreased respiration under salt stress, as they deployi their carbon reserves in the shoot for reproducing and maintaining tissue tolerance [99, 100]. Moreover, Jacoby et al [40] suggested that the varieties tolerant to salt stress allocate a lower ration of their fixed carbon into respiration, and put more into growth. According to the up-regulation of some genes related to respiration pathway including glycolysis, Krebs cycle, citric acid cycle and the electron transport chain in mitochondria in the wild type genotype, RNA-seq analysis data detected that the respiratory rate was higher in this genotype compared to the salt-tolerant mutant. Therefore, the stored energy and carbon which could be used for maintenance of plant tissue was consumed at an early time point under salinity stress in the wild-type genotype.

## Conclusions

**Fig 5:**
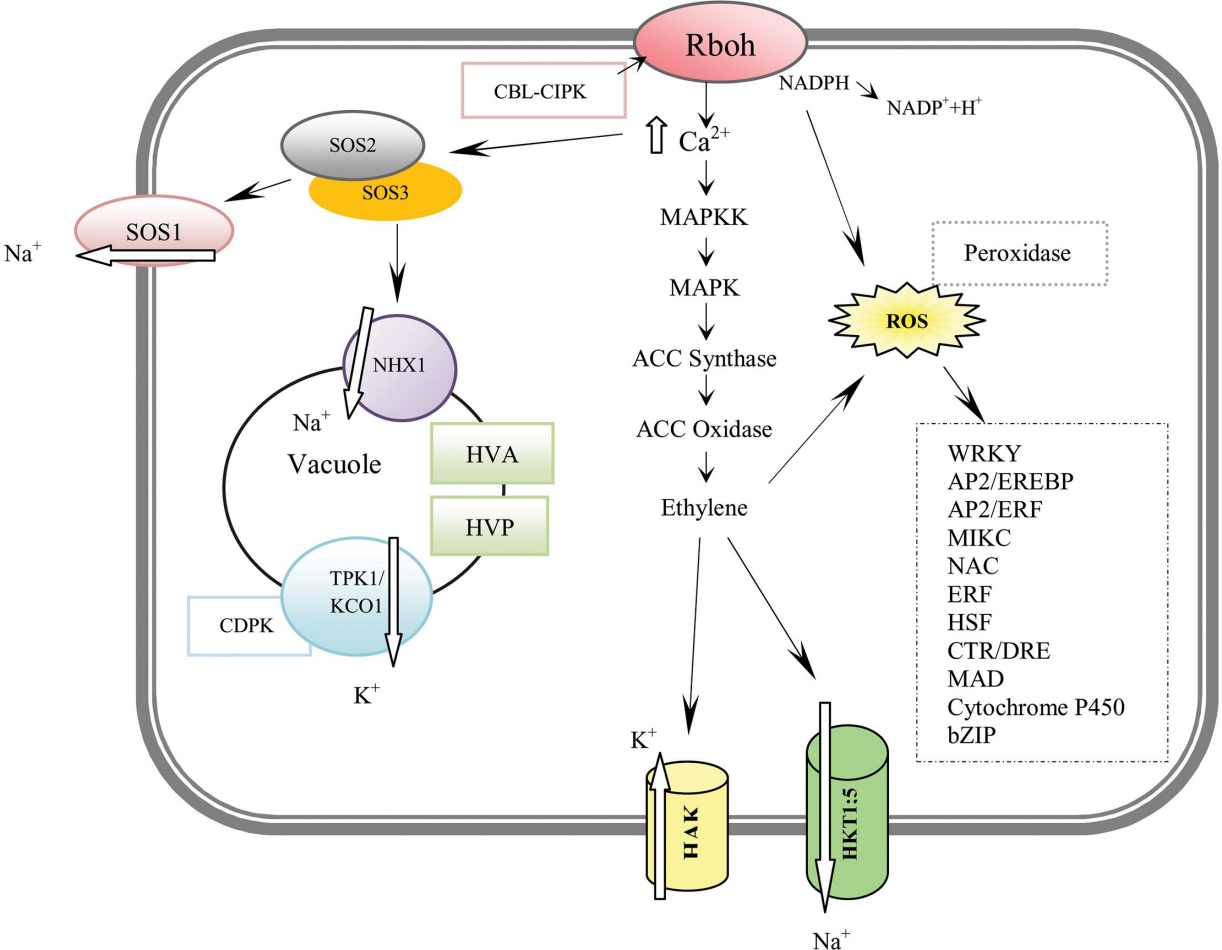
The schematic diagram of some transporters and upregulated genes in the salt-tolerant mutant genotype. It represents genes involved in salinity-stress signaling pathways and tolerant mechanisms in the salt-tolerant mutant genotype in response to 300 mM salt (six hours of exposure) in comparison to the wild-type genotype. After entrance of Na^+^ to cell cytoplasm, the activity of RBOH NADPH oxidase was increased to promote ROS formation and cytosolic Ca^2+^ content [101]. Ca^2+^ activated SOS3 and then SOS2, leading to SOS1-mediated Na^+^ extrusion [102]. On the other hand, NHX1 was activated by SOS3/SOS2 complex via HVA and HVP pumps, providing the driving force to sequester Na^+^ from cytosol to vacuole [103]. Moreover, TPK1/KCO1 is an important vacuolar permeable channel for K^+^ extrusion from vacuole to cytoplasm contributing to homeostasis regulation. Salt stress magnifies the activity of protein kinases in MAP kinase pathway to produce ethylene [104]. Ethylene can promote K^+^ retention through K^+^ channels, such as HAK and HKT to regulate Na^+^ and K^+^ homeostasis [15].

It may be concluded that the salt-tolerant mutant genotype utilized the ionic transporters and channels to control cellular homeostasis, promoting tolerance to salt stress via maintenance of cell homeostasis in contrast to its wild-type genotype. Some important ionic transporters and channels involved in complex pathways of salt-tolerant mutant genotype in the short term (six hours after the exposure to 300 mM NaCl**)** compared to the wild-type genotype re shown in Fig 5. Therefore, the salt-tolerant mutant genotype regulate cell homeostasis via a higher expression levels of some important salt-responsive genes in contrast to its wild-type genotype. The results totally indicated that the salt-tolerant mutant genotype had a better response at both the osmosis and the ionic phases under salt stress, enduring less damage in comparison to its wild-type genotype. Moreover, the comparisons demonstrated that RNA-Seq is an efficient method for analysis of genes with differential expression involved in response to salinity and identification of key biological processes for plants breeding. These technique may be also prove helpful in improvement of salt stress tolerance in other important cereal crops along with classical methods such as backcross breeding and/or new methods such as gene transformation.

## Supporting information

S1 Fig The GO category enrichment analysis for DEGs using AgriGO (v 2.0) based on molecular function for the upregulated DE transcripts in the mutant genotype under salt stress. (TIF)

S2 Fig The GO category enrichment analysis for DEGs using AgriGO (v 2.0) based on biological process for the upregulated DE transcripts in the mutant genotype under salt stress. (TIF)

S3 Fig The GO category enrichment analysis for DEGs using AgriGO (v 2.0) based on cellular component for the upregulated DE transcripts in the mutant genotype under salt stress. (TIF)

S4 Fig The GO category enrichment analysis for DEGs using AgriGO (v 2.0) based on molecular function for the upregulated DE transcripts in the wild-type genotype under salt stress. (TIF)

S5 Fig The GO category enrichment analysis for DEGs using AgriGO (v 2.0) based on biological process for the upregulated DE transcripts in the wild-type genotype under salt stress. (TIF)

S6 Fig The GO category enrichment analysis for DEGs using AgriGO (v 2.0) based on cellular component for the upregulated DE transcripts in the wild-type genotype under salt stress. (TIF)
