# Supplementary figures and images for: The RNA-seq transcriptomic analysis reveals genes mediating salt tolerance through rapid triggering of ion transporters in a mutant barley
Source: PLoS One. 2020 Mar 18;15(3):e0229513. doi: 10.1371/journal.pone.0229513 (PMC7080263; doi:10.1371/journal.pone.0229513)

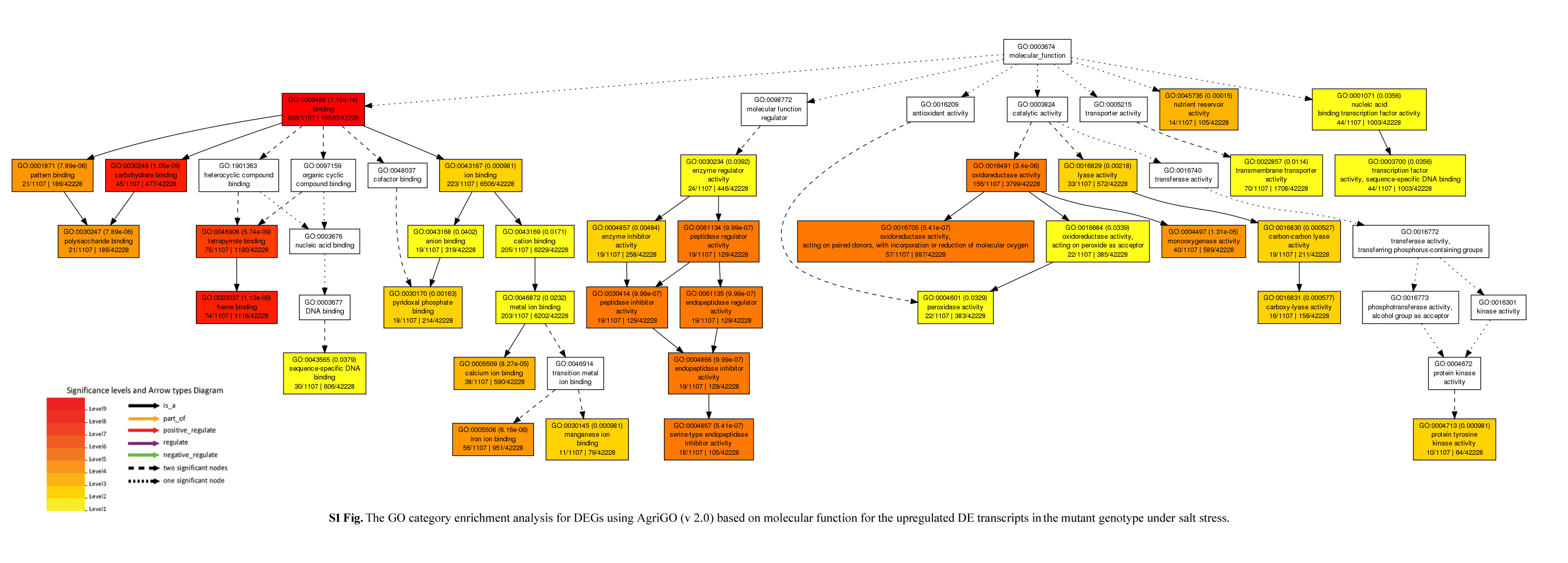

Supplement: S1 Fig — (TIF) [file pone.0229513.s001.tif]

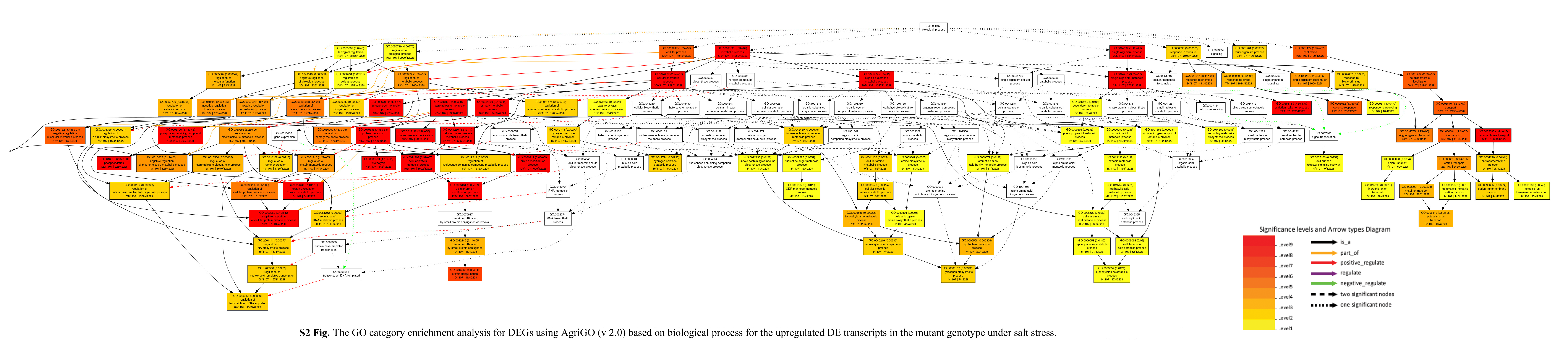

Supplement: S2 Fig — (TIF) [file pone.0229513.s002.tif]

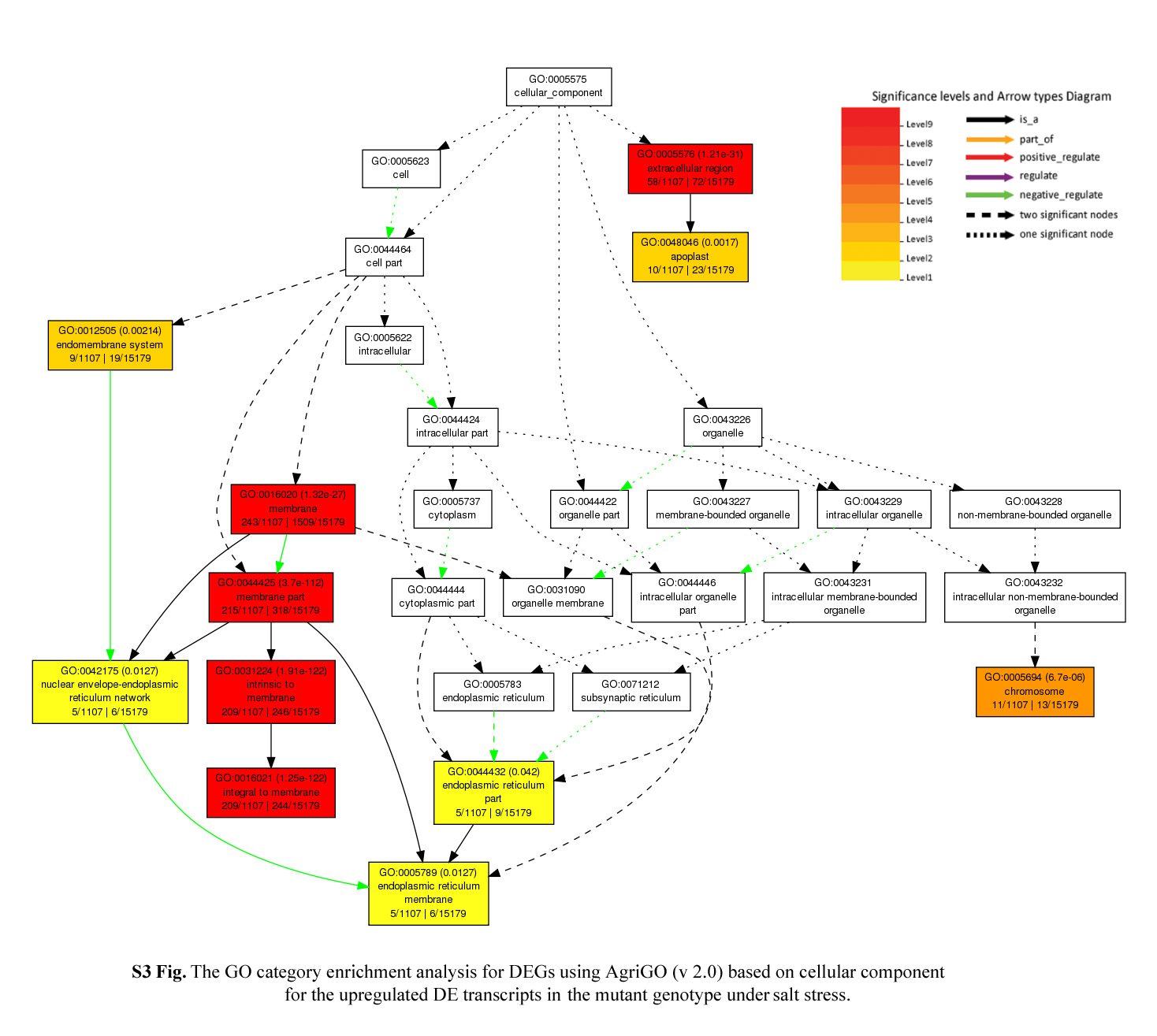

Supplement: S3 Fig — (TIF) [file pone.0229513.s003.tif]

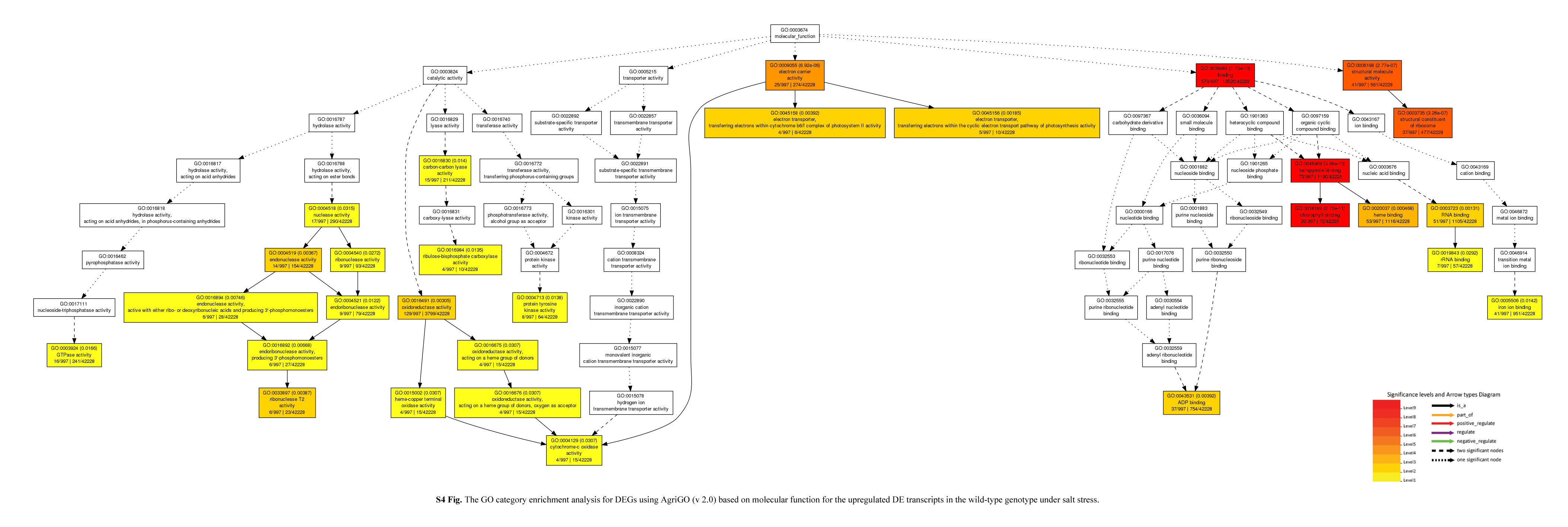

Supplement: S4 Fig — (TIF) [file pone.0229513.s004.tif]

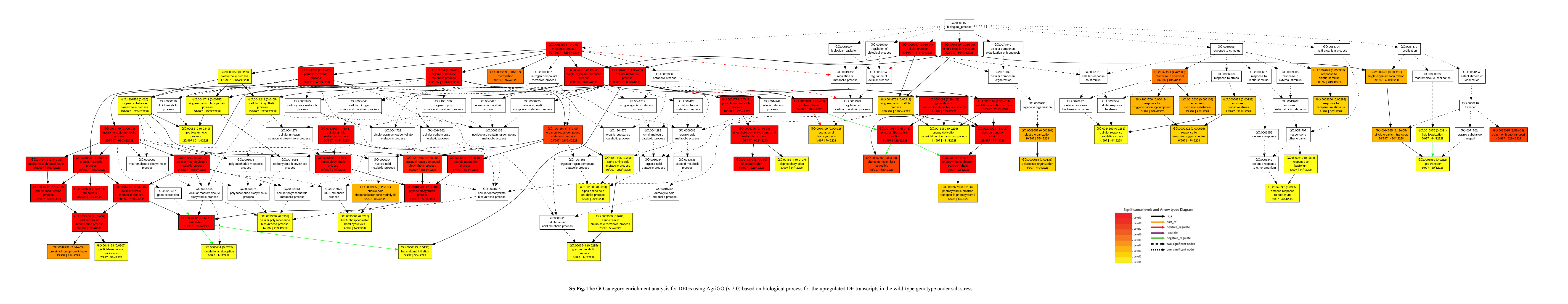

Supplement: S5 Fig — (TIF) [file pone.0229513.s005.tif]

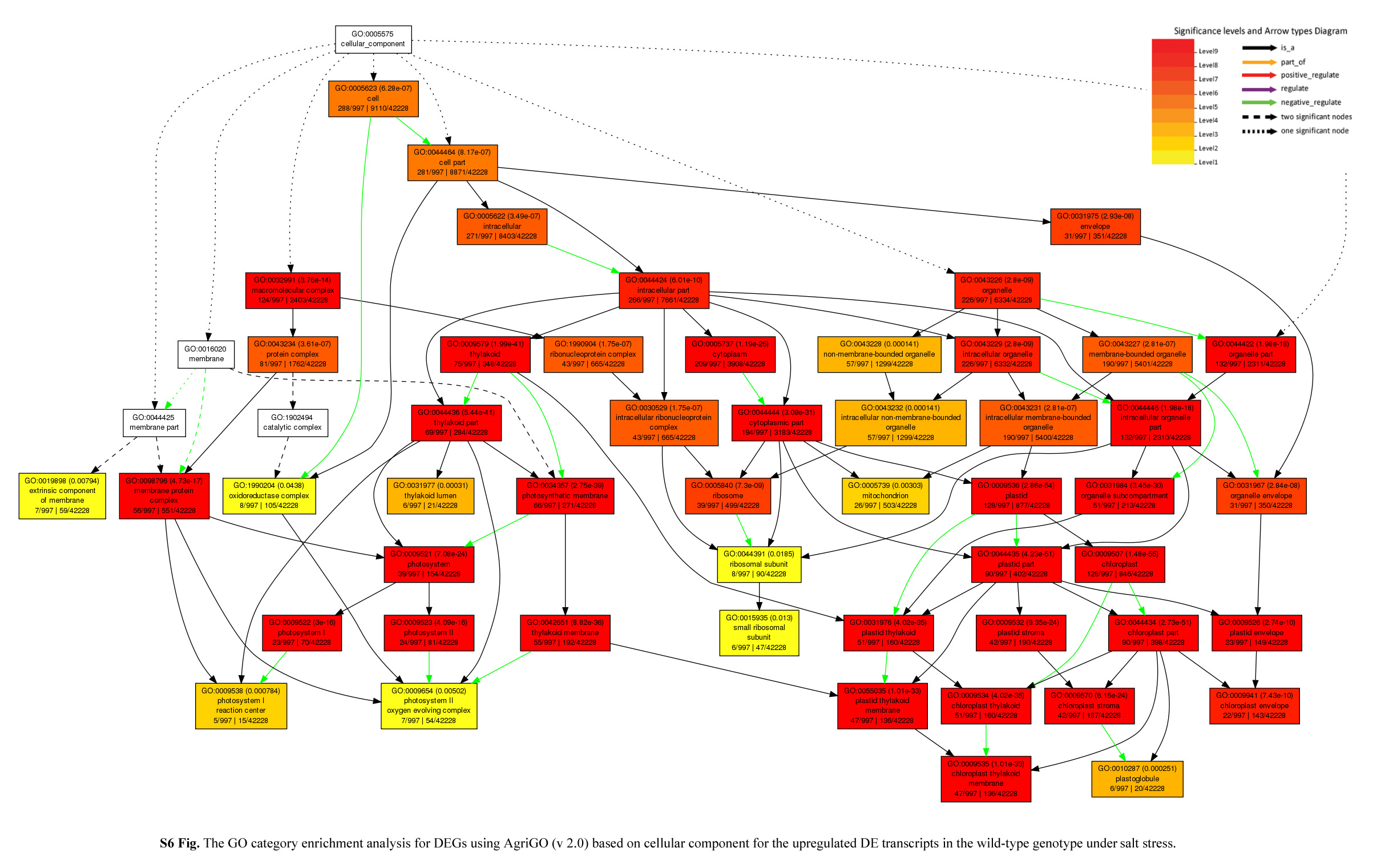

Supplement: S6 Fig — (TIF) [file pone.0229513.s006.tif]
